# Adjuvant Effect of *Lactobacillus paracasei* in Sublingual Immunotherapy of Asthmatic Mice

**DOI:** 10.3390/ph17121580

**Published:** 2024-11-24

**Authors:** Dhafer Alwayli, Xiaoli Jiang, Jiaxu Liang, Syed Rafiq Hussain Shah, Atta Ullah, Mohammed F. Z. Abusidu, Wen Shu

**Affiliations:** 1 Department of Pathogen Biology and Microecology, School of Basic Medical Sciences, Dalian Medical University, Dalian 116044, China; dhafer21@dmu.edu.cn (D.A.); xiaoly1109@163.com (X.J.); 18754663572@163.com (J.L.); syedrafiq223@gmail.com (S.R.H.S.); attaakhan902@gmail.com (A.U.); 2 Department of Biotechnology, School of Basic Medical Sciences, Dalian Medical University, Dalian 116044, China; mohammadabuseedo@gmail.com

**Keywords:** allergic asthma, house dust mite, probiotic, sublingual immunotherapy

## Abstract

**Background**: Sublingual immunotherapy (SLIT) has shown promise in mitigating allergic asthma symptoms; nevertheless, its high dose and prolonged duration of treatment raise safety concerns. This study explored the potential of *Lactobacillus paracasei* (*L. paracasei*) to enhance the effectiveness of SLIT in a mouse model of allergic asthma. **Methods**: Allergic asthma was induced in Balb/c mice following sensitization and challenge with a house dust mite (HDM) allergen. Subsequently, the mice were subjected to SLIT (66 and 132 µg) either alone or in combination with *L. paracasei* supplementation. Asthma-associated parameters, including rubbing frequency, IgE level, cytokine profiles, and histological changes, were evaluated to assess treatment efficacy. **Results**: mice that received SLIT 132 µg combined with the probiotic (combined 132) demonstrated a significant reduction in allergic symptoms (rubbing). This treatment strategy led to a marked IgE and eosinophil level decrease in serum; an increase in anti-inflammatory cytokines like IFN-γ and IL-10; and a reduction in pro-inflammatory cytokines IL-17 and TNF-α. The combination therapy also mitigated lung inflammation and supported the restoration of the structural integrity of the colon, promoting the recovery of goblet cells and mucus secretion. Probiotic treatment alone also effectively reduced IgE levels, increased IFN-γ, and decreased levels of IL-17 and TNF-α. **Conclusions**: The adjuvant effect of *L. paracasei* in enhancing SLIT represents a promising approach for improving asthma treatment efficacy.

## 1. Introduction

Asthma is a complex and chronic lung condition affecting nearly 300 million people globally, with an additional 100 million cases increasing by 2025 [1]. Allergic asthma, the most common phenotype of the disease, is featured by chronic inflammation and airway hyperresponsiveness (AHR), which can remarkably impair life quality [1,2,3]. If severe, cases of allergic asthma can be fatal and besides its link to health problems, it also contributes to significant economic burdens, reducing productivity and impacting education and quality of work [3]. Key characteristics of allergic asthma involve persistent airway inflammation, AHR, and structural changes such as airway remodeling, which collectively contribute to airflow obstruction and worsen the symptom’s severity [4,5].

The disturbed balance between various T-helper (Th) cell subtypes acts crucially in allergic asthma pathogenesis [6]. Specifically, a skewed Th2/Th1 balance is one of the key drivers of allergic asthma. Th2 cells produce cytokines such as IL-4, IL-5, and IL-13, which are pivotal in promoting IgE synthesis and subsequent histamine release (IL-4 and IL-13), eosinophilia (IL-5), and mucus oversecretion, airway hyperresponsiveness, and remodeling (IL-13). In contrast, Th1 cells typically act to counter-regulate Th2 responses through the production of IFN-γ. The Th2 pathway is overactivated in allergic asthma, whereas Th1-mediated responses are suppressed. This leads to persistent inflammation and worsened symptoms [7]. Furthermore, the development of allergic asthma is also influenced by the Th17/regulatory (Treg) imbalance. Th17 cells contribute to neutrophilic inflammation and remodeling through their production of IL-17. Increased levels of IL-17 have been linked to severe, steroid-resistant asthma. In contrast, Treg cells, which secrete anti-inflammatory cytokines like IL-10 and TGF-β, are in charge of preserving immunological tolerance and preventing excessive inflammatory responses. Treg cells are in charge of maintaining immune tolerance and averting overly inflammatory reactions because they emit anti-inflammatory cytokines including TGF-β and IL-10. A decrease in Treg cells or their malfunction exacerbates the inflammatory response, resulting in uncontrolled Th2 and Th17 activity and emphasizing the necessity of immunological balance-restoring treatment approaches [8].

Allergen immunotherapy (AIT) is one of the allergic asthma symptom modulators that involve gradually exposing patients to increasing doses of the offending allergen [9,10,11]. AIT can reduce allergic reactions through mechanisms that antagonize allergic asthma [12]. Among the various forms of AIT, sublingual immunotherapy (SLIT) has shown great potential in promoting tolerance to HDM in humans [13,14,15], and mice [16,17,18,19]. The primary mechanisms of SLIT involve the stimulation of allergen-specific IgG production by B cells and plasma cells. These IgG antibodies either compete with IgE for binding sites or prevent IgE cross-linking, thus inhibiting mast cell and basophil degranulation and the release of histamine [9,10,11,20,21]. Additionally, SLIT promotes the induction of tolerogenic dendritic cells (tDCs), which drive the production of Tregs and Bregs [22,23,24,25,26]. Despite the benefits, a SLIT high-dose and long-term treatment regimen (need 2–3 years) poses risks, including potential adverse reactions during treatment (particularly for children).

Probiotics have been demonstrated to play a substantial role in normalizing immune responses [27,28,29], though the exact mechanisms remain under investigation. Studies in murine models have shown that probiotics can trigger a shift from a Th2- to a Th1-dominant immune response, enrich T regulatory cells (Tregs), and restrict Th17 activity [30,31,32]. This immunomodulatory effect of probiotics has been associated with reducing allergic asthma in humans and mice [33,34]. Notably, *Bifidobacterium* and *Lactobacillus* augment short-chain fatty acids (SCFAs) like butyrate and acetate, which alleviate inflammation by enhancing Tregs and reducing the Th2-mediated allergic reactions [35,36]. While animal studies provide promising evidence of probiotics’ role in reducing airway inflammation and potentially preventing allergic asthma, clinical trials in humans have produced mixed results [37,38]. The variability in results may be attributed to the use of different strains of probiotics across studies [39]. Because *L. paracasei* has been proven as safe in previous research [40,41,42], we used it in this study. Additionally, this strain has shown promising results and safety in earlier studies conducted in our lab utilizing OVA-sensitized rats and mice models. This study aims to explore the adjuvant effect of *L. paracasei* in SLIT of asthmatic mice. Overall, probiotics may rebalance immune response and reduce inflammation via IL-10 induction and SCFA production, which could complement SLIT by abolishing its side effects. Therefore, some synergistic effects are expected when probiotics are simultaneously administered during SLIT, and due to their favorable safety profile, probiotics could mitigate the side effects often associated with SLIT.

## 2. Results

### 2.1. Combined 132 Ameliorates Allergic Symptoms in Mice

**Figure 1:**
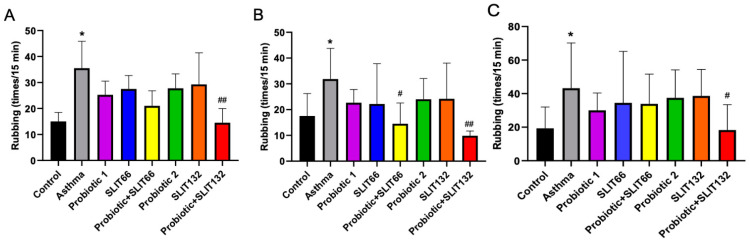
The effects of a probiotic, SLIT, and their combination on the frequency of nasal rubbing. Treatment with combined 132 demonstrated a significant decrease in the frequency of nasal rubbing on (**A**) day 19, (**B**) day 21, and (**C**) day 24 following an HDM challenge. * *p* < 0.05 vs. the control group; # *p* < 0.05 and ## *p* < 0.01 vs. the asthma group.

Nasal scratching is largely associated with histamine release. In this experiment, we established an allergic asthma model in mice and evaluated the inhibitory effect of treatments on nasal itching by applying different treatments and counting the number of rubs in the different groups of mice within 15 min post-challenge on days 19, 21, and 24. HDM-induced allergic asthma mice displayed significantly increased frequencies of rubbing compared with control mice (Figure 1A–C; *p* < 0.05). As shown in Figure 1A–C, the number of rubs for allergic asthma mice given combined132 treatment was lower than the number for those not given treatment (*p* < 0.01, *p* < 0.01, and *p* < 0.05, respectively). These results suggested that combined 132 ameliorated the symptoms of rubbing in allergic asthma mice.

### 2.2. Changes in Serum IgE and Cytokine Levels After Treatment with SLIT and L. paracasei

**Figure 2:**
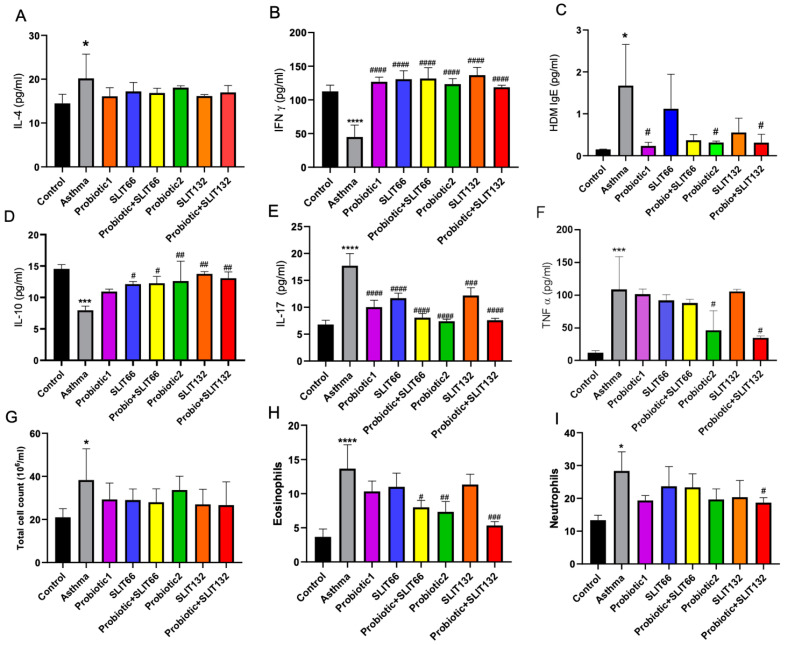
The effects of a probiotic, SLIT, and their combination on inflammatory cell count, IgE, and cytokine concentrations. Serum IgE and cytokines of asthmatic and treated mice of all groups were measured by ELISA. The concentrations of (**A**) pro-inflammatory cytokine IL-4, (**B**) Interferon gamma (IFN-γ), (**C**) HDM IgE, (**D**) IL-10, (**E**) IL-17, (**F**) tumor necrosis factor alpha (TNFα). The enumeration of inflammatory cells in blood, including (**G**) total blood cells, (**H**) eosinophils, and (**I**) neutrophils obtained from various groups. * *p* < 0.05, *** *p* < 0.001, and **** *p* < 0.0001 vs. the control group; # *p* < 0.05, ## *p* < 0.01, ### *p* < 0.001, and #### *p* < 0.0001 vs. the asthma group.

To assess the effectiveness of the probiotic, SLIT, and their combination on HDM-induced allergic asthma in mice, various immune parameters, including serum IgE and cytokine levels (IL-4, IL-17, TNFα, IFN-γ, and IL-10), were detected using ELISA. As depicted in Figure 2A–I, HDM sensitization and subsequent challenge led to a significant elevation in the levels of IgE and IL-4, IL-17, and TNF-α when compared to the control group. On the other hand, the levels of IFN-γ and IL-10 were notably reduced in these HDM-exposed groups, indicating a clear immunological response consistent with HDM sensitization. After treatment application, IFN-γ levels showed a significant increase across all treatment groups (Figure 2B; *p* < 0.0001). IL-17 levels were significantly reduced following treatment, with the most notable reduction observed in the probiotic-only and combined groups (Figure 2E; *p* < 0.0001). The production of IL-10 was significantly elevated in the probiotic-only, SLIT 132, and combined 132 groups compared to the asthma group (*p* < 0.001) (Figure 2D). TNF-α levels also significantly decreased in both the probiotic-only and combined 132 groups (*p* < 0.05). IgE levels were markedly reduced following treatment with probiotic-only and combined 132 groups (*p* < 0.05). Although a downward trend in serum IL-4 levels was observed in the combined 132 group, the reduction was not statistically significant (Figure 2A).

### 2.3. Combined 132 Reduces Eosinophil and Neutrophil Levels in Mice Serum

Figure 2G,H show the total and differential inflammatory cells in serum for neutrophils and eosinophils. According to the results, there were no significant differences among the total cell counts in any of the treatment groups vs. asthma (Figure 2G). However, the evaluation of the differential cell counts for neutrophils and eosinophils revealed a percentual decrease in eosinophils and neutrophils in the combined 132 group compared to the asthma group (Figure 2H,I, *p* < 0.001 and *p* = 0.05, respectively).

### 2.4. Combined 132 Mitigates Lung Damage and Reduces Airway Inflammation in HDM-Sensitized Mice

**Figure 3:**
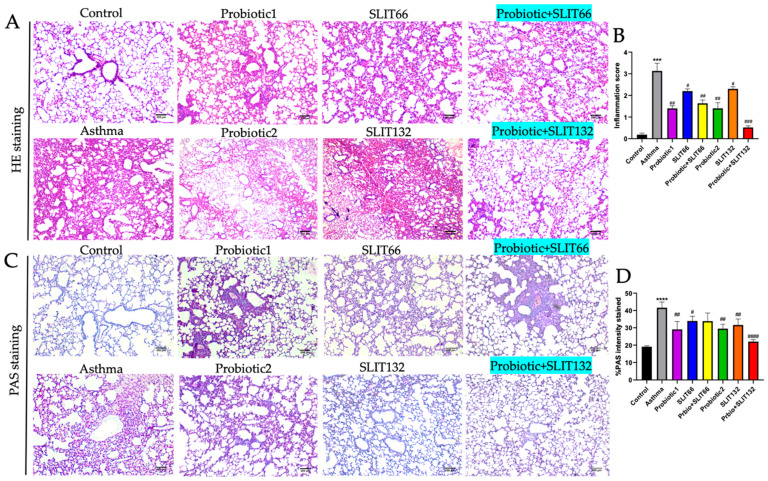
A probiotic, SLIT, and their combination improved asthma-induced histopathological change in mice lungs. (**A**) H&E staining of mice lung tissues. Lung sections stained with H&E revealed infiltrations of inflammatory cells in HDM-sensitized and -challenged mice, contrasting with the control group where such aggregations were absent. Notably, treated mice exhibited distinctive features in this context. (**B**) Inflammation score. (**C**) PAS staining was applied to mouse lung sections following intranasal HDM exposure. (**D**) % PAS intensity of stained tissue. Scale bar = 200 μm; Magnification: 40×. *** *p* < 0.001 and **** *p* < 0.0001 vs. the control group; # *p* < 0.05, ## *p* < 0.01, ### *p* < 0.001, and #### *p* < 0.0001 vs. the asthma group.

Histopathological assessments were conducted to further evaluate the degree of HDM-induced airway inflammation. Asthmatic mice displayed significant immune cell infiltration in lung tissues. As shown in Figure 3A, all treatments exhibited less lung inflammation and cell infiltration; however, treatment with combined 132 notably further improved outcomes. Inflammation in the lung tissue’s peribronchial and perialveolar areas was graded using a subjective scale from 0 to 4. After treatment with combined 132, the inflammation score decreased significantly, approaching levels similar to those of the control group (Figure 3B, *p* < 0.001). To examine interstitial GC hyperplasia, we utilized PAS staining. The asthma group displayed a higher number of PAS-positive cells compared to the control group. However, treating asthmatic mice with combined 132 significantly reduced the area of PAS-stained and PAS-positive cells (Figure 3C,D, *p* < 0.0001).

### 2.5. Combined 132 Improves GCs’ Number and Mucin Production in the Colon of HDM-Sensitized Mice

**Figure 4:**
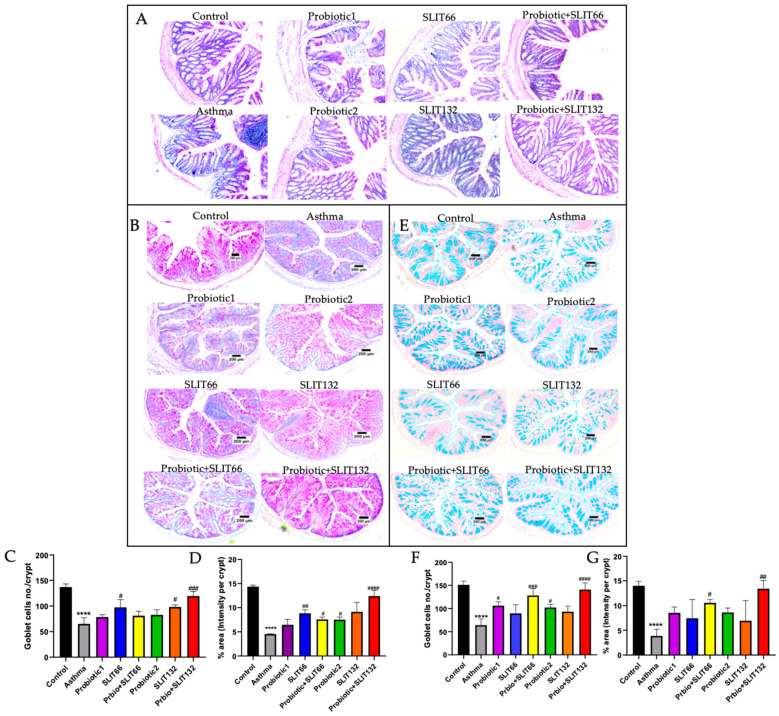
Microscopic examinations of colon tissues. (**A**) H&E staining of a transversely cut colon section; (**B**) colon sections stained with PAS; (**C**) the number of GCs/crypt; (**D**) % PAS-stained area of GCs and mucin; (**E**) colon sections stained with AB staining; (**F**) the number of GCs/crypt in AB-stained areas; and (**G**) % AB-stained areas were assessed in all groups. Magnification: 100×. **** *p* < 0.0001 vs. the control group; # *p* < 0.05, ## *p* < 0.01, ### *p* < 0.001, and #### *p* < 0.0001 vs. the asthma group.

We here identified whether asthma in mice could lead to colon injury and possible improvement after treatment application. In the asthma group, mice exhibited apparent changes in the colon histostructure, with a damaged and unevenly arranged epithelium after staining with H&E. The mucosal space appeared to be ill defined, and the infiltration of inflammatory cells was observed (Figure 4A). To further assess the effect of asthma on goblet cells and mucin content within the colon, we conducted AB and PAS staining. This technique stains acidic mucins blue using AB and neutral mucins magenta with PAS. PAS and AB staining of the asthma lung revealed hallmarks of pathological features of allergic asthma, including a decrease in GCs’ number, and intensity of acid mucin production (Figure 4B,E). The total mucin intensity and the number of GCs in colonic tissue sections were measured by ImageJ software (version 1.4.3.67) Figure 4C,D,F,G). However, GCs’ number and mucin secretion were recovered after staining with PAS (*p* < 0.001 and *p* < 0.0001) and AB (*p* < 0.0001 and *p* < 0.01), and inflammatory infiltration was reduced following treatment, particularly with combined 132.

## 3. Discussion

Our study involved the administration of sublingual allergen therapy, a strategy rooted in prior research showing its potential in modulating T-cell responses and inhibiting eosinophil-mediated inflammation. This method, particularly useful in pediatric populations due to its non-invasive nature, avoids the need for frequent clinical visits or injections [43]. However, the extended treatment typically lasts two to three years—and the gradual increase in dosage can lead to undesirable side effects, potentially reducing patient compliance and carrying concerns about its overall safety [44,45]. Recent advancements have focused on enhancing the efficacy of SLIT while mitigating its side effects. Our investigation aimed to enhance immunity and reduce inflammation through the combined use of probiotics and SLIT, with the expectation that effective regulation can be achieved safely even at low doses and over a short duration.

It is well understood that SLIT follows a dose- and time-dependent relationship, though these aspects remain subjects of ongoing research and discussion [46]. In this study, we implemented two dosage levels: 66 µg (low dose) and 132 µg (relatively higher). Despite the difference, both doses still fall within the low-dose range when compared to previously established SLIT dose standards. The SLIT regimen alone shows poor efficacy in our short-term model; this is consistent with other studies [16]. It is inspiring that the combination therapy exhibited a significant reduction in rubbing counts, indicating a pronounced alleviation of allergy-associated symptoms, which is two-fold lower in dose and 11-fold shorter in duration than other studies that demonstrated beneficial effects [47,48]. This is in agreement with the hypothesis of this study; interestingly, our low-dose SLIT in a short time showed a similar effect and less side function compared to high-dose SLIT in a long time when combined with the probiotics. The efficacy of SLIT may be potentiated with higher doses and prolonged treatment durations, though this can induce a higher incidence of adverse reactions. Moreover, using low doses with longer duration gives conflicting results in some studies. While some showed a good effect [16], others showed a weak one [49].

The significant effects observed in the combined group are based on the restoration of immune balance. This combination treatment strategy led to a marked IgE, eosinophil, and neutrophil level decrease in serum; an increase in anti-inflammatory cytokines like IFN-γ and IL-10; and a reduction in pro-inflammatory cytokines IL-17 and TNF-α. It has better improvement in TNF-α, eosinophils, and IgE than the SLIT group. IgE is the key target for AIT therapy [9,10,11]; it can serve as a characteristic indicator for evaluating the level of therapies. The lack of a significant reduction in IgE indicates that low-dose, short-term SLIT has limited efficacy in treating asthma. Supporting this, Shima et al. also found that SLIT did not significantly lower IgE but still managed to reduce eosinophilic inflammation [18]. This outcome underscores the potential synergistic role of probiotics in supporting the overall efficacy of SLIT. IL-17 and TNF-α cytokines are essential in neutrophil infiltration within the lungs of asthma patients, and their decline indicates an attenuating of neutrophilic inflammation [50]. Previous studies reported that IL-17 and TNF-α are acting synergistically during the inflammation process [51]. Thus, downregulating these cytokines partially approves combination efficacy. Additionally, the better recovery of eosinophils and neutrophils suggests that the combined group more effectively regulated immune imbalance, thereby achieving better therapeutic outcomes. Moreover, it should be pointed out that the probiotic similarly triggered certain important parameters, i.e., a significant production of IL-10 and decrease in IL-17 levels, and these responses could have helped the promotion of SLIT through pathways mediated by cell signals. We suggest that our probiotic appears consequently to be a probiotic strain that induces, in immature DC, the production of IL-10 concomitant with a low-level induction of IL-17. While the combination therapy did not significantly impact IL-4 levels, it significantly elevated IFN-γ levels, further enhancing the anti-inflammatory effect of the treatment.

Lung inflammation can cause airway epithelial barrier dysfunction and remodeling [52]. It has been known that Th2-secreted cytokines such as IL-4 and IL-5 can release IgE and eosinophil migration to airways (airway eosinophilia), mucus hypersecretion, and GC hyperplasia [53]. In our study, asthma mice displayed inflammatory cell infiltration (both alveolar and peribranchial) as HE- and PAS-stained cells are increased in intensity as shown with a high purple-red color and, with the administration of combination 132, could markedly alleviate inflammation as displayed with the lowest inflammation score compared with other treatment regimens. This complies with other studies’ findings [16,17]. This airway barrier injury is most likely related to our immunological findings. Relatedly, we could also analyze colon histopathology to further explore whether asthma has an impact on the intestine, particularly, the colon. Undeniably, intestinal epithelial cells are crucial for maintaining normal intestinal permeability and barrier integrity. Mucins, GC-secreted materials, play a role in maintaining the epithelial barrier [54]. The examination of colon photomicrographs showed a decrease in GCs along with a lower PAS staining intensity in the asthma group compared to the normal control group, implying a lower secretion of neutral mucins. Similar findings have been observed in previous studies where it was seen that asthma induces intestinal injury [55]. While different treatment groups provide variable significant effects in this study, most superior improvements were observed with the combination of SLIT and a probiotic. The combination may lead to an early onset of SLIT efficacy through the additive effects of the probiotic activation of Treg cells in the lung and intestine. However, SCFAs can be a contributor to these effects, which require further exploration as agreed on by other studies [56,57]. In our study, combined 132 group treatment restored the damaged mucosal integrity caused by asthma by replenishing the GC population, implying a favorable modulation of the immune response with the combined treatment.

In summary, we found that the combined treatment of a probiotic with SLIT demonstrated a synergistic effect in reducing inflammation and ameliorating asthma, attenuating the airway and colon barrier primarily in a gut–lung axis-dependent manner and microbiota-dependent modulation. Probiotic treatment alone also showed some level of protection against asthma. The superiority of the SLIT–probiotic combination was most likely clear, potentially because the SLIT mechanism of action is similar to that of a probiotic. However, these results remain contentious, and further research is needed to deeply understand these synergistic effects, including the need for human validation and more profound and comprehensive immunological investigations in mice.

## 4. Materials and Methods

### 4.1. Mice, Housing, and Grouping

Six-to-eight-week-old male BALB/c mice (*n* = 80), weighted 18–20 g, were supplied by Liaoning Changsheng Biotechnology Co., Ltd. (Dalian, China). Mice were cohoused (5/cage) in autoclavable polypropylene cages with free access to chow and water under specific pathogen-free conditions, standard temperature conditions, and humidity with a 12 h light–dark cycle. Mice, who had 7 days of co-acclimatization, were blindly distributed into eight groups (*n* = 10/group), as described in Table 1.

### 4.2. Probiotic Culture Conditions

*L. paracasei* (Department of Pathogen Biology and Microecology, Dalian Medical University) was cultured in sterile Lactobacillus MRS broth (Hopebio, Qingdao, China) at 37 °C for 24–48 h under anaerobic conditions. Cell counts were determined by plating serial dilutions, and the optical density was measured for the subsequent cell number count in the next culture. After being centrifuged for 10 min at 4 °C, 6000× *g* rpm, the harvested probiotic was washed thrice with PBS and then freshly administered.

### 4.3. Asthma Model Sensitization and Challenge Protocol

**Figure 5:**
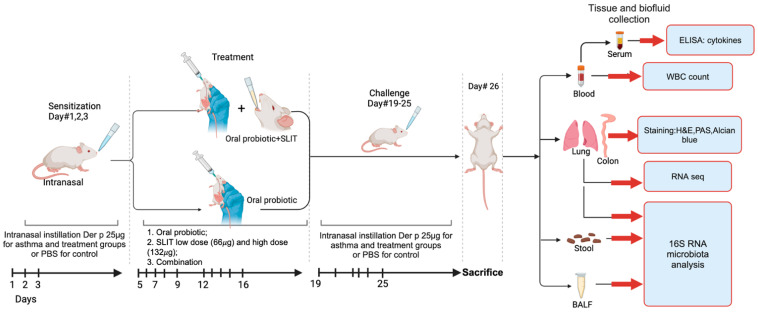
Experimental protocol design of allergic asthma with HDM and *L. paracasei* treatment. Mice were intranasally sensitized to HDM Der p extract (25 µg Der p in 10 µL PBS) for 3 days. SLIT was administered for 5 consecutive days/week for 2 weeks, as either high dose (132 µg/day) or low dose (66 µg/day), followed by 6 days of intranasal challenge exposure (same dose with sensitization). Unlike SLIT HDM-treated groups, control and asthma and probiotic-treated mice received PBS for SLIT.

To establish the allergic asthma mouse model, HDM-derived protein Der p powder (*Dermatophagoides (D.) pteronyssinus*, Greer Labs, Lenoir, NC, USA), with a dry weight of 16.7 mg, was first dissolved in 25 mL PBS (Solarbio, Beijing, China) at a final concentration of 6.68 μg/μL. Our asthma model protocol was adopted from a previous one [58] with some modifications. Briefly, mice were given 25 μg HDM extract intranasally on sensitization days 1, 2, and 3 in 20 μL sterile PBS. On days 19–25, mice were challenged with HDM (i.n. 25 μg in 20 μL PBS). PBS (20 μL) was administered in the control group. On day 26, mice were sacrificed (Figure 5).

### 4.4. SLIT and Probiotic Intervention Protocol

Mice were treated sublingually with either 10 μL PBS alone (control and asthma group), 66 μg HDM extract (SLIT66 alone and Probio + SLIT66 group), or 132 μg HDM extract (SLIT132 alone and Probio + SLIT132 group). Mice received SLIT on day 5 then continued for five days in a row for two weeks with two-day intervals as rest days. In the low-dose group, *L. paracasei* oral intake was performed throughout the experiment days, whereas in the high-dose group, *L. paracasei* was administered only during SLIT days, as depicted in Figure 5. A daily dose of 1.0 × 10^9^ colony-forming units of *L. paracasei* (in 200 μL of PBS) was given intratracheally to each mouse.

### 4.5. Symptom Sign Evaluation

The number of nasal and facial rubbing induced by HDM stimulation was counted by three observers without prior knowledge of the groups’ identity within 15 min immediately after the allergen challenge. The effects of *L. paracasei* and SLIT instillation on the frequency of nasal rubbing after HDM re-exposure at days 19, 21, and 24 were assessed.

### 4.6. Blood Cytopathological Analysis

The mice were killed on day 26, after which the blood (approximately 0.5–1 mL) was collected from the vena cava vein, placed in a labeled vial containing EDTA, and analyzed for total and differential white blood cell counts. Total cell numbers and numbers of eosinophils and neutrophils were counted for all groups.

### 4.7. Enzyme-Linked Immunosorbent Assay (ELISA)

Blood was taken from the mice vena cava using a 1 mL syringe. To obtain serum, samples were incubated at room temperature for 1 h, centrifuged at 8000× *g* at 4 °C for 15 min, and then transferred to new tubes. The IgE concentration and serum cytokines IL-4, TNF-α, IL-17, IFN-γ, and IL-10 were also measured by ELISA (Quanzhou Ruixin Biotechnology Co., Ltd., Quanzhou, China), according to the manufacturer’s instructions.

### 4.8. Tissue Histoprocessing and Staining

After sacrificing the animals, the left lungs and colons of mice were collected and preserved in 4% paraformaldehyde (Seven Biotech, Beijing, China) and sent to a histology lab for paraffin embedding and cutting into 4 μm sections. The sections were rehydrated after deparaffinization and stained with Hematoxylin and Eosin (H&E), Periodic Acid Schiff (PAS), and Alcian Blue (AB) (Solarbio, Beijing, China) as instructed by the kit’s protocol to investigate airway and gut pathological and inflammatory processes in the mice. To quantify and evaluate the degree of histopathological inflammation and mucus production, lung and colon sections were examined with a microscope equipped with a camera (McAudi Industrial Group Co., Ltd., Xiamen, China) and images were analyzed with Image J software (version 1.4.3.67). The lung inflammation score was measured as previously described [59]. Briefly, a five-point scoring method was used to determine the inflammatory score: normal = 0, a few cells = 1, one layer of a cell ring = 2, two to four layers of a cell ring = 3, and more than four layers of a cell ring = 4. Using the ImageJ program, the PAS and AB % area stained, as well as the GCs’ number and colon crypt, were identified and examined.

### 4.9. Statistical Analysis

With GraphPad Prism 8 (GraphPad, San Diego, CA, USA), all statistical analyses were carried out using either the one-way analysis of variance (ANOVA) or the *t*-test (for comparisons between two groups). The findings were presented in the form of the mean ± standard deviation (SD). When *p*-values were less than 0.05, the results were considered statistically significant.

## 5. Conclusions

The comprehensive examination of the study purpose, investigating the synergistic impact of combining probiotics with a higher dose of sublingual HDM immunotherapy in an asthma mouse model, yielded notable and promising outcomes. The study findings provide compelling evidence that the combination of probiotics with a high dose of sublingual HDM immunotherapy in an asthma mouse model not only effectively reduces allergic symptoms but also demonstrates immunomodulatory and tissue-protective effects. It is noteworthy that short-term probiotic treatment alone also showed some level of protection against asthma. However, these results remain controversial and need to be confirmed in human studies and more profound and comprehensive immunological investigations in mice. These results suggest a promising avenue for improving therapeutic outcomes while alleviating potential side effects and shortening the duration of treatment.

## Figures and Tables

**Table 1:** The mice grouping of the study.

Group No.	Group Name	Explanation
Group I	Control	Intranasal (i.n.) 20 μL phosphate-buffered saline (PBS) + 10 μL PBS SLIT
Group II	Asthma	i.n. 25 μg HDM in 20 μL PBS +10 μL PBS SLIT
Group III	Probiotic1	HDM asthma model + 10 μL PBS SLIT + *L. paracasei* (day1–end of model)
Group IV	SLIT66	HDM asthma model+ SLIT 66 μg
Group V	Probiotic + SLIT66	HDM asthma model + (*L. paracasei* + SLIT66 μg combination)
Group VI	Probiotic2	HDM asthma model + 10 μL PBS SLIT + *L. paracasei* (day 5–16)
Group VII	SLIT132	HDM asthma model+ SLIT 132 μg
Group VIII	Probiotic + SLIT132	HDM asthma model + (*L. paracasei* + SLIT132 μg combination)

## Data Availability

The raw data supporting this study will be made available by the corresponding author upon request.
